# Editorial: Insights in female reproductive longevity

**DOI:** 10.3389/fendo.2025.1556565

**Published:** 2025-02-05

**Authors:** Takashi Minegishi

**Affiliations:** Gunma University, Maebashi, Japan

**Keywords:** median randomaization, AMH, PCOS, inhibin, inflamation

It is well known that female reproduction ability decreases around the ages of 40 due to age-related change; however, in recent years, the number of women who are over the age of 40 and trying to conceive has increased remarkably.

Follicular development is a highly complex process regulated by multiple factors in gonadotropin-independent and dependent phases. The growth and maturation of the ovarian follicle requires the coordinate function of somatic cells and the oocyte. Crosstalk between the oocyte and the microenvironment is mediated by direct contact with surrounding cells, the extracellular matrix, and signaling molecules, including hormones, growth factors, and metabolic products.

In this section, AMH and inhibin were picked up and discussed as a useful biomarker and targets for the multiple pregnancy. Anti-Müllerian hormone (AMH), an indirect indicator of the number of remaining follicles, is clinically used as a test for ovarian reserve. Typically, a decline suggests a decrease in the number of remaining follicles in relation to ovarian toxicity caused by interventions, which may implicate fertility. In contrast, serum AMH levels are elevated in patients with polycystic ovary syndrome. AMH is produced primarily in the granulosa cells of the preantral and small antral follicles. Thus it varies in association with folliculogenesis and the establishment and shrinking of the follicle cohort. Ovarian activity during the female half-life, from the embryonic period to menopause, is based on folliculogenesis and maintenance of the follicle cohort, which is influenced by developmental processes, life events, and interventions (Iwase et al.).

Here, the current study aimed to investigate the potential role of gene immunization against INH on immune responses, follicle development, serum reproductive hormone (FSH, E_2_, and P_4_) concentrations, and evaluate the reproductive effect of this vaccine on estrous, ovulation, and conception rates in beef cattle (Meng et al.).

Furthermore, controlling chronic low-grade inflammation in the ovaries may be a novel therapeutic strategy to improve the ovarian microenvironment and minimize the loss of oocyte quality. Unhealthy maternal conditions such as aging, polycystic ovary syndrome (PCOS), and endometriosis can directly hamper the follicle microenvironment during folliculogenesis and reduce oocyte quality. Common to aging, polycystic ovary syndrome, and endometriosis may be that chronic low-grade inflammation induces oxidative stress and subsequent tissue stiffness in the ovaries. Inflammaging (inflammatory aging) is a chronic and mild inflammatory condition associated with aging. Senescent cells secrete inflammatory substances called senescence-associated secretory phenotype factors that induce oxidative stress and cause chronic inflammation in surrounding cells. In PCOS and endometriosis, inflammatory cytokine levels are elevated in the follicular fluid. In PCOS, ovarian androgen excess and insulin resistance promote low-grade inflammation in the follicle microenvironment. In endometriosis, iron overload induces chronic inflammation and oxidative stress, leading to a new type of cell death, ferroptosis. Controlling chronic low-grade inflammation in the ovaries may be a novel therapeutic strategy to improve the ovarian microenvironment and minimize the loss of oocyte quality (Orisaka et al.).

In clinical aspect, a powerful epidemiological method known as Mendelian randomization (MR) can be used to clarify the causality between ANM(abnormal age at natural menopause) and other diseases or traits. The present review describes MR studies that included ANM as an exposure, outcome and mediator. The findings of MR analyses on ANM have revealed that higher body mass index, poor educational level, early age at menarche, early age at first live birth, early age at first sexual intercourse, and autoimmune thyroid disease appear to be involved in early ANM etiology (Zhang et al.). Another group developed and validated a nomogram based on five ovarian reserve indicators to predict the risk of retrieving fewer than 10 oocytes at one oocyte retrieval cycle in women ≤ 35 years of age. The model demonstrated good discrimination and calibration, indicating its reliability for clinical application. This nomogram offers a practical and accurate tool for early identification of young women with potentially decreased ovarian reserve, enabling timely intervention and personalized management strategies (Liu et al.).

